# Heterologous expression in *E. coli* reveals that bicarbonate transporter BicA2 mediates carbon uptake in marine *Prochlorococcus* spp.

**DOI:** 10.1093/ismejo/wrag222

**Published:** 2026-08-27

**Authors:** Loraine M Rourke, Caitlin S Byrt, G Dean Price, Benedict M Long

**Affiliations:** Plant Science Division, Research School of Biology, Australian National University, 134 Linnaeus Way, Acton, ACT 2601, Australia; Loraine M. Rourke, Plant SynBio Australia, Plant Science Division, Research School of Biology, 134 Linnaeus Way, Australian National University, Acton, ACT 2601, Australia; Plant Science Division, Research School of Biology, Australian National University, 134 Linnaeus Way, Acton, ACT 2601, Australia; Plant Science Division, Research School of Biology, Australian National University, 134 Linnaeus Way, Acton, ACT 2601, Australia; Plant Science Division, Research School of Biology, Australian National University, 134 Linnaeus Way, Acton, ACT 2601, Australia; Discipline of Biological Sciences, School of Science, University Drive, The University of Newcastle, Callaghan, NSW 2308, Australia; ARC Centre of Excellence in Synthetic Biology, School of Science, University Drive, The University of Newcastle, Callaghan, NSW 2308, Australia

**Keywords:** BicA, bicarbonate transport, CO_2_-concentrating mechanism, cyanobacteria, HCO_3_^−^, *Prochlorococcus*sodium-dependent transport, uptake kinetics

## Abstract

The widespread oceanic cyanobacterial *Prochlorococcus* genus is a major contributor to global carbon fixation, yet mechanisms enabling this lineage to elevate intracellular inorganic carbon as a substrate for photosynthesis remain unresolved. Cyanobacterial CO_2_-concentrating mechanisms typically rely on membrane-bound bicarbonate (HCO_3_^−^) transporters SbtA1, SbtA2, BicA and BCT1, and CO_2_-to-HCO_3_^−^ conversion uptake systems (CO_2_ pumps; NDH-I_3_ and NDH-I_4_), to elevate a cellular HCO_3_^−^ pool for use by Rubisco-containing carboxysomes. Evidence suggests *Prochlorococcus* harbours carboxysomes with a low-CO_2_-specificity Rubisco, implying a functional CCM dependent on active HCO_3_^−^ uptake. However, canonical CO_2_ pumps are absent, leaving distant HCO_3_^−^ transporter homologues, BicA2 and SbtA2, as prime candidates for HCO_3_^−^ transport in this group. Yet these have not been functionally characterized. Here we demonstrate that BicA2 from *P. marinus* CCMP1375 mediates Na^+^-dependent HCO_3_^−^ uptake in *Escherichia coli*, whereas BicA2 from *P. marinus* CCMP1986 is inactive in its native form but acquired transport function through a single amino acid substitution during adaptive laboratory evolution. These findings confirm BicA2 as a low-affinity, Na^+^-dependent bicarbonate transporter with variable flux, revealing a previously uncharacterized CCM component in *Prochlorococcus*. This mechanistic insight reshapes our understanding of carbon acquisition strategies in the most abundant photosynthetic organism on Earth and highlights evolutionary plasticity in transporter function with implications for global biogeochemical cycles.

## Introduction

*Prochlorococcus* spp., along with oceanic *Synechococcus* spp. of the α-cyanobacterial sub-group [1], are attributed with an estimated 25% of annual net CO_2_ fixation in marine environments [2]. *Prochlorococcus* are the smallest and most abundant single-celled photosynthetic organism [3, 4]. They have some of the smallest genomes of all photosynthetic organisms [5], yet they are genetically diverse, with ~50% of the pangenome conserved across species [6]. The remainder of the genome is quite diverse, most likely due to the variation in environmental niches in which they grow, including high-light (typically surface-water) and low-light (typically deep-water) adapted ecotypes [6].

**Figure 1:**
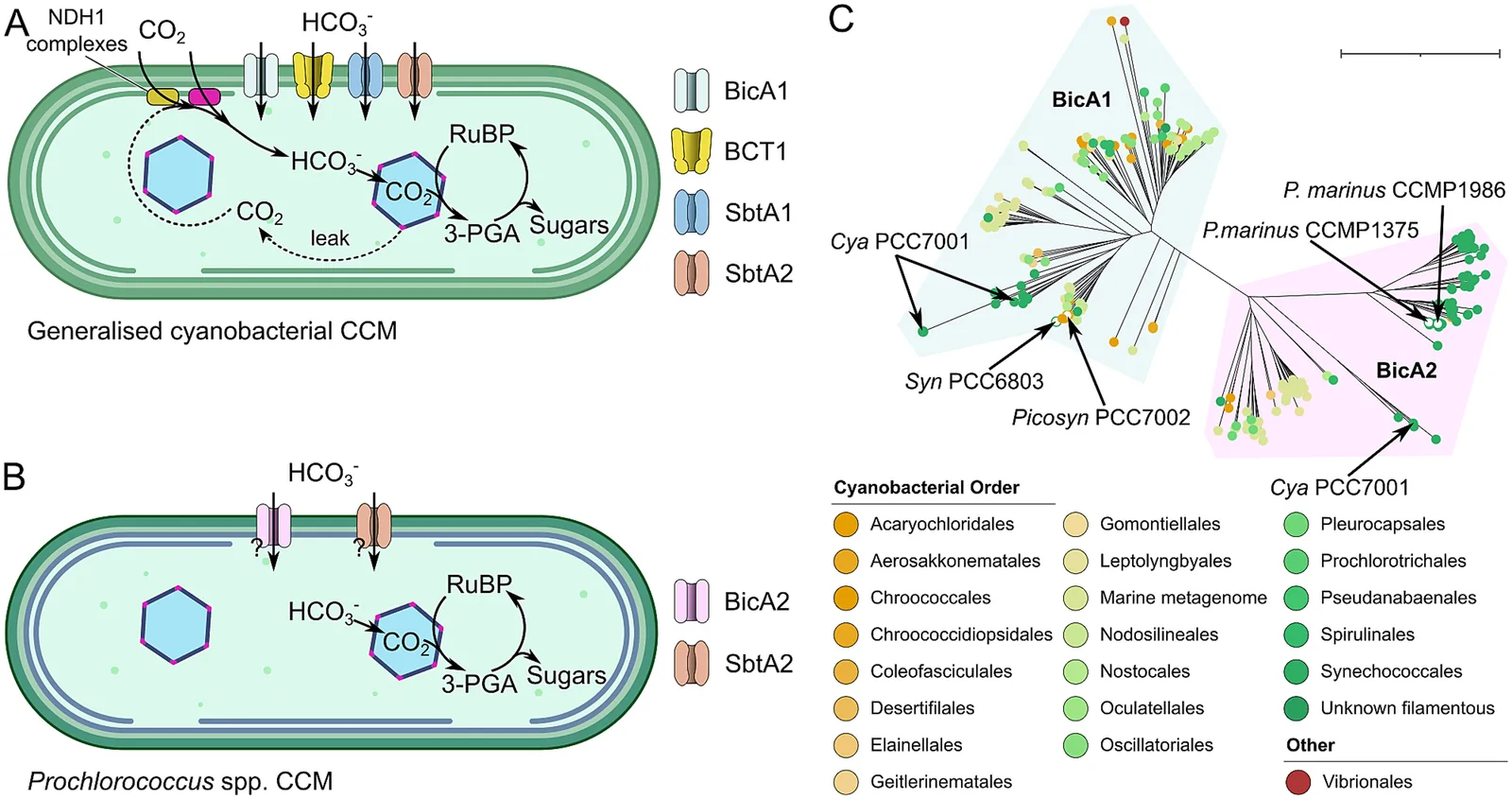
Inorganic carbon (Ci) transport in generalized cyanobacterial CO_2_ concentrating mechanism (CCMs) and *Prochlorococcus* spp. (A) CO_2_ concentrating mechanism components in most cyanobacteria is generalized to be driven by inorganic carbon (Ci) transport by up to four characterized bicarbonate transporters (BicA1, BCT1, SbtA1, SbtA2; [15–17, 19, 48, 57]) and two CO_2_ to HCO_3_^−^ conversion systems [NDH-1 complexes using ChpX or ChpY; 11, 58] on the thylakoid membranes. These collectively supply a large cellular HCO_3_^−^ pool that serves the Rubisco-containing carboxysome, where a specialized carbonic anhydrase enables elevation of CO_2_ concentrations for rapid CO_2_ fixation. (B) In *Prochlorococcus* spp., genes coding for members of the SbtA2 [19] and putative BicA2 transporters are proposed to enable efficient CCM function [29]. (C) Phylogenetic analysis of BicA proteins from cyanobacteria reveal two BicA families [BicA1 and BicA2; 26]. Each is a member of the larger SulP transporter family [27]. Members of the BicA1 clade from *Picosynechococcus* PCC7002 (*Picosyn* PCC7002) and *Synechocystis* PCC6803 (*Syn* PCC6803; both identified in the BicA1 clade as white circles with coloured outline) have been previously characterized to show HCO_3_^−^ transport function [17]. Reported in this study is a functional analysis of BicA2 proteins from *Prochlorococcus* CCMP1375 and *Prochlorococcus* CCMP1986 (identified in the BicA2 clade as white circles with coloured outline). Multiple genes for BicA1 and BicA2 proteins from *Cyanobium* PCC7001 (*Cya* PCC7001) are identified. Sequence alignment performed using MAFFT 7.526 [38] from a curated BicA dataset containing 223 protein sequences [39] with default parameters and 1000 bootstrap replicates. Protein sequences were recovered from the NCBI non-redundant protein database and accession numbers are provided in the associated online dataset. Tree graphics were generated using iTol [40]. Tree scale is 0.1 substitutions per amino acid.

Efficient inorganic carbon (Ci) accumulation to drive photosynthesis in cyanobacteria and other phytoplankton (e.g. diatoms and green micro-algae) is primarily attributed to their CO_2_-concentrating mechanisms (CCMs) that support high rates of photosynthetic CO_2_ fixation [7, 8]. The CO_2_ concentration is increased in the immediate vicinity of the site of the CO_2_-fixing-enzyme, ribulose-1,5-bisphosphate carboxylase/oxygenase (Rubisco), which converts CO_2_ to sugars for net carbon gain [7, 9, 10]. As a key element of their CCMs, cyanobacteria have several CO_2_ and HCO_3_^−^ transporters located on the thylakoid and plasma membranes (Fig. 1), respectively, which actively accumulate bicarbonate (HCO_3_^−^) into their cells [11]. This elevated HCO_3_^−^ pool then diffuses into protein-encapsulated microcompartments called carboxysomes [7, 12] where carboxysomal carbonic anhydrase (CA), co-located with Rubisco, interconverts HCO_3_^−^ and CO_2_, increasing the available CO_2_ concentration for rapid carboxylation [13, 14].

Currently, cyanobacterial CCMs broadly have a total of six identified inorganic carbon (Ci; CO_2_ and HCO_3_^−^) transport systems (Fig. 1), classified into two main types; those that transport HCO_3_^−^ from outside the cell (BicA1, SbtA1, SbtA2, BCT1) [15–19] as well as specialized CO_2_-to-HCO_3_^−^ conversion systems (NDH-I_3_ and NDH-I_4_) that are located on internal thylakoid membranes [20–23]. Cyanobacterial HCO_3_^−^ transporters play a fundamental role in carbon assimilation in aquatic environments, since HCO_3_^−^ is the dominant form of Ci available in seawater and it has relatively slow diffusion across biological membranes compared with CO_2_ [24, 25]. Both HCO_3_^−^ and CO_2_ uptake systems are found in most cyanobacterial species, except for *Prochlorococcus* spp*.* which do not possess genes for the known CO_2_-to-HCO_3_^−^ conversion systems [26, 27]. In addition, *Prochlorococcus* spp. generally only possess SbtA2 homologues and distant relatives of BicA1, which have been termed BicA2 [20, 26, 28]. SbtA2 was recently described as an apparent Cl^−^/SO_4_^2−^-dependent HCO_3_^−^ transporter when SbtA2 proteins from *Synechococcus* spp. were heterologously expressed in *E. coli* [19]. However, the same study showed that SbtA2 homologues from *Prochlorococcus* spp. were inactive in this system, such that their physiological role remains uncertain. Despite the significance of this genus in global primary productivity [2, 4] and evidence of CCM function [29, 30], there has been no functional characterization of Ci transport in *Prochlorococcus* spp. to date, leaving the mechanism of inorganic uptake in this important group unresolved.

Here we describe the functional characterization of several BicA2 transporters from the globally dominant *Prochlorococcus* clade to confirm candidate proteins involved in HCO_3_^−^ uptake. *Prochlorococcus* spp. *bicA2* genes were expressed in *E. coli* and screened for transport activity. BicA2 from the high-light ecotype *P. marinus* CCMP1986 showed no activity in its native form; however, adaptive laboratory evolution [15] produced a variant with a single point mutation that transported HCO_3_^−^ in *E. coli*. Comparative sequence analysis of this mutated, functional form of BicA2 highlighted conserved amino acid sequence motifs near this mutation, guiding the identification of a BicA2 from the low-light ecotype *P. marinus* CCMP1375, which exhibited Na^+^-dependent HCO_3_^−^ transport in *E. coli*. These findings provide direct evidence that a *Prochlorococcus* membrane protein can mediate bicarbonate transport, revealing a candidate pathway for carbon entry into the biosphere.

## Materials and methods

### *bicA2* gene cloning and expression construct assembly

*bicA2* from *P. marinus* CCMP1986 (hereafter *bicA2–1986*) was amplified by PCR (Phusion High-Fidelity DNA Polymerase Thermo Fisher Scientific) from genomic DNA using primers listed in Table S1 and cloned (with and without mycH6 tag) using type IIS restriction enzyme Loop Assembly [31, 32], under the control of the tetracycline inducible/repressible promoter and the *rrnB* terminator in the pCk1 (Kanamycin resistant) vector. *bicA2* from *P. marinus* CCMP1375 (hereafter *bicA2–1375*) and its variants were synthesised by Synbio Technologies (USA) and cloned similarly to *bicA2–1986*, except the *lacI^q^-trc* promoter was used for expression experiments due to initial tests showing this was a more reliable promoter for this protein. Construct sequences were verified using Sanger sequencing (Macrogen Inc, Seoul, South Korea).

### HCO_3_^−^ uptake experiments using silicone oil centrifugation-filtration

HCO_3_^−^ uptake was examined in *E. coli* DH5α expressing putative transporters along with appropriate controls as per [33] with the following modifications. Starter cultures were grown in liquid LB overnight from glycerol stock (BicA2–1375 and variants) or from LB + 1.5% agar that had been growing for 72 h at 22°C (BicA2–1986 and variants). These cultures were then diluted 1:5 into fresh LB and grown at 37°C for ∼1.5 h to an OD600 of ~0.6. Expression was induced by the addition of isopropyl β-D-1-thiogalactopyranoside (IPTG) to a final concentration of 1 mM, or anhydrotetracycline hydrochloride (aTc; Merck) to 400 nM. Cells were depleted of Ci by washing three times with 1 ml of CO_2_-free buffer (20 mM BisTrisPropane-H_2_SO_4_ pH 7.5 + 200 mM NaCl +10 mM K_2_HPO_4_). Uptake was initiated by addition of NaH^14^CO_3_ (∼60 MBq mmol^−1^) to a final concentration of 0.5 mM for 30 s over a 50 μl silicone oil mix (AR200:20 3.5:4), above a 20 μl kill solution (3 M sodium hydroxide, 50% methanol) in a 400 μl polypropylene tube (Beckman Coulter) [33]. Following rapid centrifugation, accumulated H^14^CO_3_^−^ in the cell pellet was quantified by liquid scintillation counting of the cell pellet resuspended in 2 M NaOH as described [33]. Due to the density of the silicone oil used, the maximum concentration of NaCl assessed in this assay was 200 mM. Assay buffer composition was varied as indicated to assess ion dependence.

### Complementation of CO_2_-dependent CAfree *E. coli*

*E. coli* CAfree strain [34] containing *bicA2* gene expression plasmids were grown overnight in LB + 20 mM HEPES-KOH pH 8.0 with antibiotics at 37°C in air supplemented with 4% CO_2_. These were diluted 1:5 in LB + 20 mM HEPES-KOH pH 8.0 with antibiotics and grown for ~60 min. Culture densities (OD_600_) were measured and standardized before spot-plating onto Luria–Bertani (LB) agar containing ~170 mM NaCl, 1.5% Bacto Agar and 20 mM HEPES-KOH pH 8.0, supplemented with kanamycin (50 μg^.^ml^−1^) and 400 μM isopropyl IPTG (for plasmids containing the *lacI^q^-trc* promoter) or 200 nM aTc [35]. Plates were incubated for 24–48 h at 37°C in air levels of CO_2_ (~0.04%) or 4% CO_2_ v/v in air.

### Membrane enrichment and WB test for tagged proteins

Samples were taken from induced *bicA2* gene expression cultures of *E. coli* used for Ci uptake experiments, pelleted and stored frozen at −20°C until membrane enrichment was performed as follows. Frozen pellets were resuspended in 0.7 ml of lysis buffer (50 mM HEPES-KOH pH 8.0, 100 mM NaCl, and 5% glycerol) supplemented with ~30 kU rLysozyme (Merck) and incubated at room temperature with inversion for 30 min. Cells were then bead-beaten for 3 min with ~200 μl glass beads at maximum speed (BioSpec mini-beadbeater-16). Cells debris was removed by centrifugation (20 238 × *g*, 30 s). Supernatant was transferred to fresh tubes and centrifuged at 16 700 × *g* for 30 min at 4°C. The supernatants (soluble fraction) were retained and pellets were resuspended overnight in 50 μl lysis buffer including 1% n-dodecyl-β-D-maltoside (Merck) at 4°C. Insoluble material in this membrane-enriched fraction was removed by centrifugation (10 min, 20 238 × *g* 4°C). Dithiothreitol (50 mM) and SDS-PAGE loading buffer were added to both soluble and membrane-enriched fractions, heated for 5 min at 90°C, and loaded onto NuPAGE 4–12% Bis-Tris sodium dodecyl sulfate-polyacrylamide gel electrophoresis gels. Separated proteins were subsequently transferred to polyvinylidene fluoride membrane (Merck-Millipore) using Transblot Turbo (BioRad). After blocking with 5% skim milk powder in Tris buffered saline, membranes were probed with anti-myc antibody (cat # M4439, Merck) and AP-conjugated-secondary antibody (cat # A3562, Merck), and developed with Attophos (Promega Corporation). Blots were visualized using the Chemidoc MP imaging system (BioRad) [36].

### Adaptive laboratory evolution

In our hands CAfree *E. coli* is unable to grow at 4% CO_2_ in M9 minimal medium unless supplemented with 1% LB. A starter culture of CAfree carrying plasmid pCk1-*bicA2–1986* was first grown from glycerol stock in LB containing 50 μg^.^ml^−1^ kanamycin at 37°C in air supplemented with 4% CO_2_ for ~18 h. This starter culture was then diluted 1:50 in 5 ml M9 medium containing 1% LB supplemented with 50 μg^.^ml^−1^ kanamycin and 50 nM aTc, and grown at 37°C in air supplemented with CO_2_ to 1%. Once the cultures grew dense overnight at these permissive CO_2_ conditions, they were diluted 1:100 and incubated in air levels of CO_2_ (0.04%) and continued as batch dilution cultures until the culture grew dense overnight. Cells from cultures that grew in air levels of CO_2_ overnight (100 μl), were plated on to LB + 1.5% agar supplemented with kanamycin and aTc, and growth was assessed in air. Ten independent isolates from air-grown plates were cultured and pDNA was prepared using miniprep spin column (Qiagen) for sequencing (Macrogen Inc, Seoul, South Korea). To ensure the mutation conferring growth in air was not due to a mutation in the background strain of CAfree, purified plasmids were reintroduced to CAfree cells and assessed for growth in air.

### BicA multiple sequence alignment and phylogenetic tree construction

Using identified amino acid sequences; GAGATMGTV and GAGATMRTV as BLAST queries [37], we recovered a total of 233 BicA protein sequences from cyanobacteria with either of these motifs conserved. MSA was performed using MAFFT 7.526 (with default parameters and 1000 bootstrap replicates) [38] after curating the BicA sequence dataset [39] to remove incomplete or duplicate sequences. Trees (Fig. 1; Supplementary Fig. S1) were visualized and edited using iTol (https://itol.embl.de/) [40]. Clade designation to either BicA1 or BicA2 was initially confirmed after visual inspection of the tree, and sequence logos were subsequently generated using separate alignments of the BicA1 and BicA2 sequences using WebLogo3 (https://weblogo.threeplusone.com/create.cgi) [41].

## Results

Phylogenetic analysis demonstrated that there are multiple BicA subfamilies across cyanobacterial clades (Fig. 1) [26, 27]. Here, we refer to the previously characterized BicA proteins [17, 18, 42] as BicA,1, and the other main family as BicA2 (Fig. 1), consistent with previous terminology [26]. Several species possess at least two BicA proteins, including *Cyanobium* PCC7001, which appears to have two BicA1 proteins and one BicA2 protein ranging between 43% and 65% sequence identity to each other (Fig. 1; Supplementary Fig. S2). Since *Prochlorococcus* possesses only BicA2 and SbtA2 proteins (Fig. 1; 29), and SbtA2 from *Prochlorococcu*s were not functional in *E. coli* [19], we considered that BicA2 from *Prochlorococcus* should display HCO_3_^−^ transport function.

To assess HCO_3_^−^ transport by candidate BicA2 proteins, we exploited a mutant strain of *E. coli*, CAfree. CAfree lacks all cellular CA enzymes [14, 15, 19, 33, 34], and is unable to survive at atmospheric CO_2_ concentrations (~0.04% v/v CO_2_ in air) due to its inability to rapidly interconvert CO_2_ and HCO_3_^−^. The latter is required for small molecule and fatty acid biosynthesis via HCO_3_^−^‐dependent carboxylases [43] and the low HCO_3_^−^ supply in CAfree under ambient CO_2_ leads to growth inhibition [15, 19]. However, CAfree can grow in elevated CO_2_ (4% v/v in air) where bulk diffusion of CO_2_, and its slow interconversion with HCO_3_^−^, leads to sufficient HCO_3_^−^ to support one-carbon metabolism and growth. CAfree can survive in air levels of CO_2_ if an active HCO_3_^−^ transporter is present in the plasma membrane [15, 16, 19, 33, 34]. The constraints of CAfree to survive in ambient CO_2_ can be exploited as a screening system to test for functional HCO_3_^−^/CO_2_ transporters and carbonic anhydrases [14–16].

**Figure 2:**
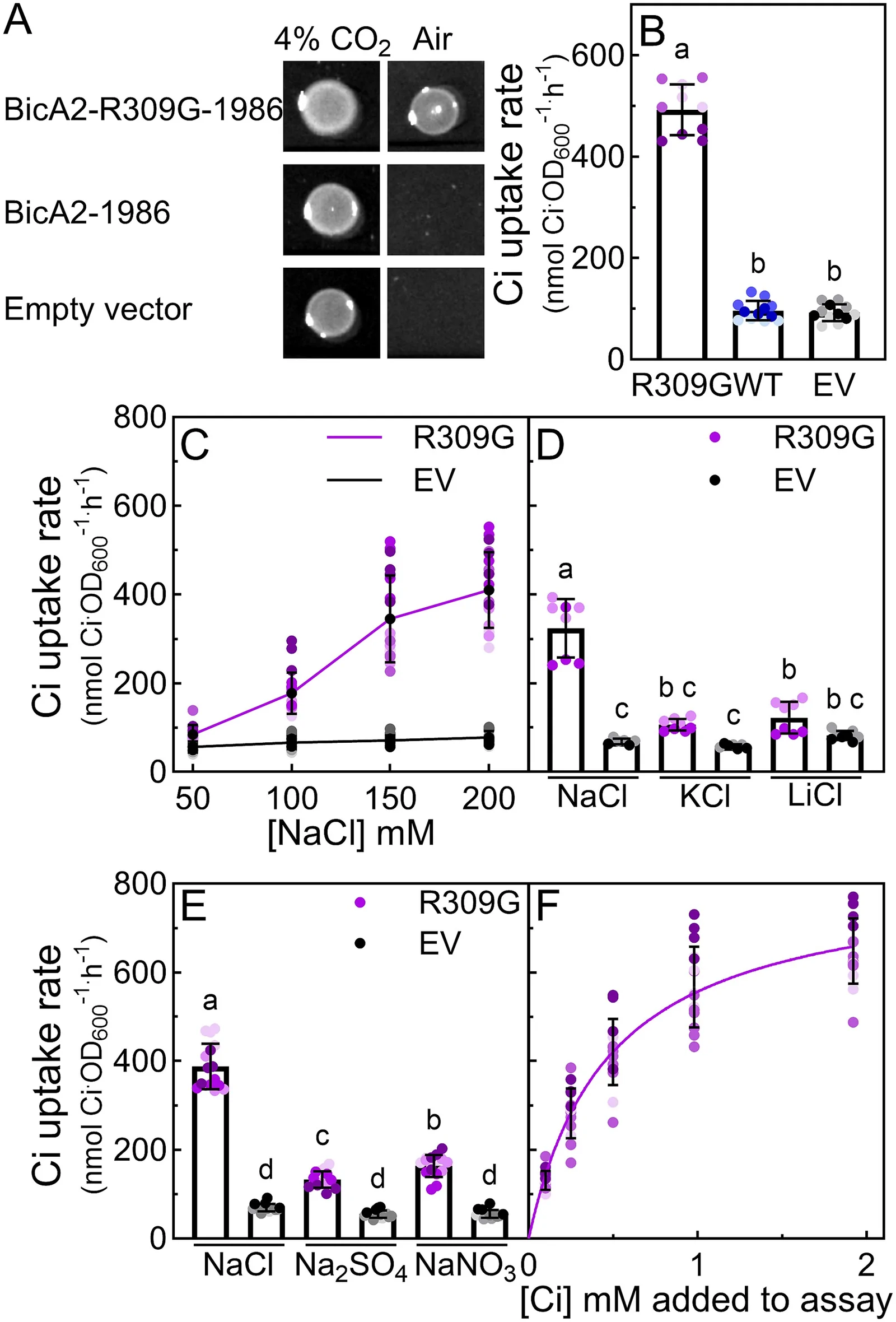
Characterization of BicA2 forms from *Prochlorococcus* CCMP1986 via CAfree *E. coli* complementation and HCO_3_^−^ uptake assays. (A) Cultures of CAfree *E. coli* expressing BicA2-R309G-1986, BicA2–1986 and empty vector were spotted onto selective medium and incubated at air or 4% CO_2_. (B) the HCO_3_^−^ uptake activity of *E. coli* DH5α expressing BicA2-R309G-1986 (R309G), BicA2–1986 (WT) and a negative control (empty vector; EV) were assessed. Assay buffer composition was 20 mM BTP-H_2_SO_4_ pH 7.5, 10 mM K_2_HPO_4_, and 200 mM NaCl. n = 10–12 samples from three biological replicates. (C) the HCO_3_^−^ uptake activity of *E. coli* DH5α expressing BicA2-R309G-1986 (R309G) and empty vector (EV) were assessed in response to increasing NaCl concentration. HCO_3_^−^ uptake was assessed across a range of NaCl concentrations (50–200 mM). Data are presented as mean ± s.d.; n = 11 samples from three biological replicates. (D) HCO_3_^−^ uptake was assessed in the presence of NaCl, LiCl or KCl. Assay buffer composition was 20 mM BTP H_2_SO_4_ pH 7.5, 10 mM K_2_HPO_4_, 10 mM Na_2_HPO_4_, with relevant salts (150 mM NaCl or 150 mM KCl or 150 mM LiCl). Data are presented as means ± SD.; n = 6–8 samples from two biological replicates. (E) HCO_3_^−^ uptake was assessed in response to different Na^+^ salts. Assay buffer composition: 20 mM Hepes pH 7.5, 10 mM K_2_HPO_4_, and each of the salts (150 mM NaCl or 75 mM Na_2_SO_4_ or 150 mM NaNO_3_). Data are presented as means ± SD different letters indicate significantly different means when assessed using one-way analysis of variance (ANOVA; *P* < 0.05) (F) the HCO_3_^−^ uptake activity of *E. coli* DH5α expressing BicA2-R309G-1986 was assessed over a range of NaHCO_3_ concentrations (0.1–2 mM) to determine the affinity for HCO_3_^−^. For kinetic parameter estimates, the empty vector rates were subtracted prior to non-linear-regression to estimate the Michaelis–Menten constants (Table 1). Data are presented as mean ± s.d. with empty vector rates for each condition subtracted; n = 14–16 samples from four biological replicates.

Expression of BicA2–1986 from *P. marinus* CCMP1986 in CAfree did not rescue cell growth in air levels of CO_2_ (Fig. 2A). To assess gain-of-function potential in this non-functional BicA2 form, we carried out adaptive lab evolution in CAfree cells under permissive CO_2_ supply [15]. Here, we have taken advantage of the naturally occurring mutation rate of *E. coli* (~1 in 10^9^–10^10^ nucleotides per generation) [44] to evolve function in a non-functional HCO_3_^−^ transporter. This was achieved by growing CAfree expressing non-functional BicA2 proteins in growth-permissive CO_2_ levels (~1% CO_2_ v/v in air). Under these conditions, populations that arose from cells with evolved transporter function then dominated the population under low CO_2_ due to a growth advantage [15, 19]. Two independent attempts to evolve function in BicA2–1986 using adaptive laboratory evolution resulted in isolates carrying a point mutation (A > G at 925 bp in the gene coding sequence) giving rise to an amino acid change, R309G. This functional form is hereafter referred to as BicA2-R309G-1986.

To characterize BicA2-R309G-1986 function, HCO_3_^−^ uptake rates were determined in *E. coli* DH5α expressing both the mutant and WT transporter forms. This was assessed using H^14^CO_3_^−^ uptake assays employing the silicone oil centrifugation-filtration method [19]. Elevated HCO_3_^−^ uptake rates were observed in *E. coli* DH5α expressing BicA2-R309G-1986 when compared to those expressing either BicA2–1986 or an empty vector control, under standard assay conditions of 200 mM NaCl (Fig. 2B). Typically, after subtraction of the empty vector control rates, HCO_3_^−^ uptake rates of ~500 nmol Ci^.^OD_600_^–1.^h^−1^ were observed in *E. coli* DH5α cells expressing BicA2-R309G-1986 (Fig. 2B and C). Mutation in the gene was confirmed as the driver of the observed function by re-cloning the mutated sequence into the original plasmid backbone and reintroducing into DH5α *E. coli* and measuring H^14^CO_3_^−^ uptake (Supplementary Fig. S3). This confirmed growth at air levels of CO_2_ was attributable to the mutated BicA2-R309G-1986 transporter.

**Figure 3:**
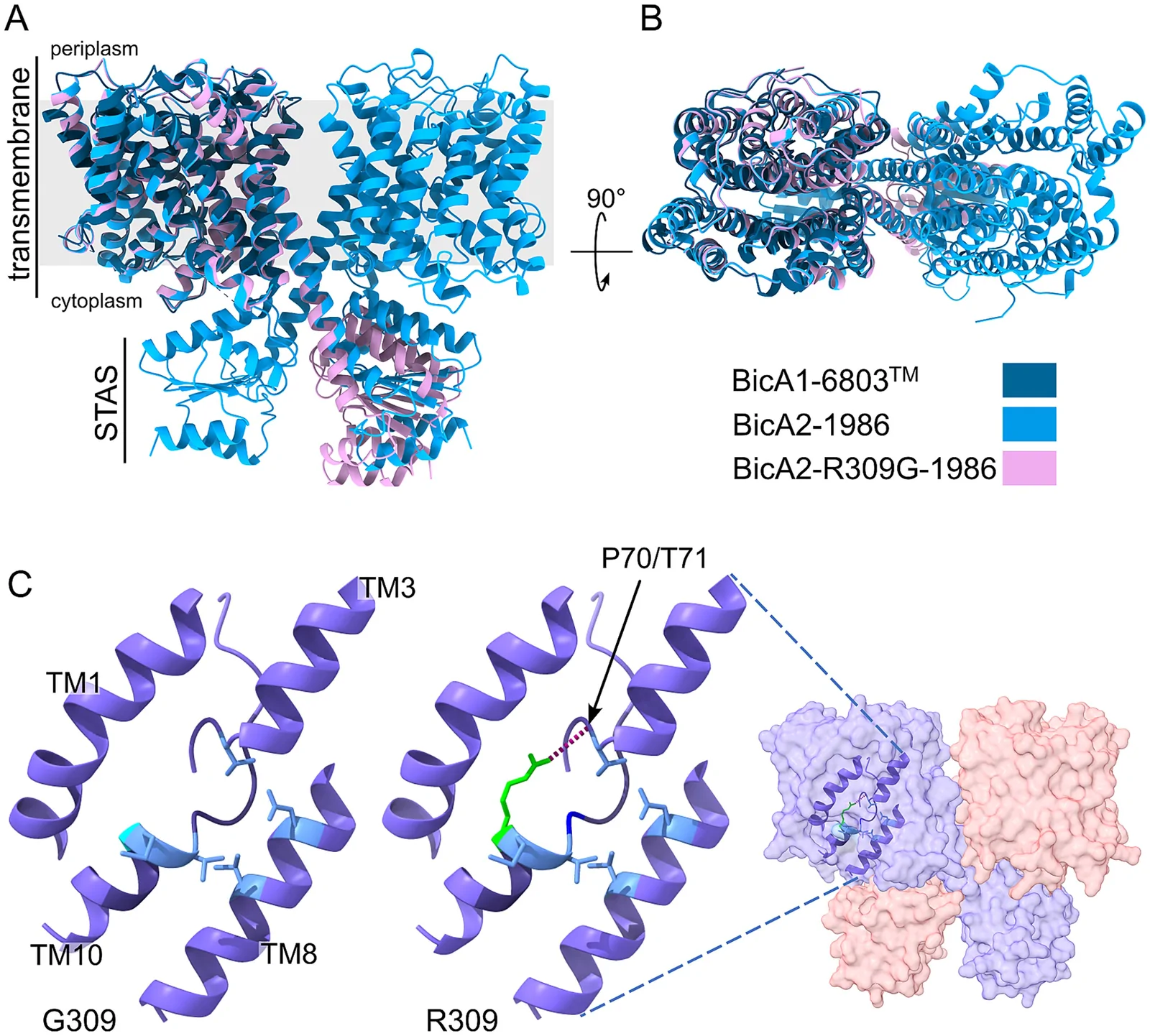
Comparison of predicted protein structures of BicA2–1986 variants and BicA1–6803™. (A) Predicted strutures of the BicA2–1986 homodimer and BicA2-R309G-1986 monomer [AlphaFold 3; 56] overlaid with the transmembrane domain of BicA1 from *Synechocystis* PCC6803 [PDB: 6KI1; BicA1–6803™; 42]. Structures are shown in side view, with shaded area indicating the approximate membrane boundary. The transmembrane and regulatory sulfate transporter anti-sigma antagonist (STAS; [42, 46]) domains are indicated. (B) Extracellular view of the structural alignment shown in panel A. (C) Predicted substrate-binding region of BicA2–1986 showing residue 309 within the conserved GAGATM[G/R]TV motif of transmembrane helix 10 (TM10). The active G309 form (left) and inactive wildtype R309 form (right) are shown. Putative HCO_3_^−^ identified in BicA1 [42] and conserved in BicA2 are highlighted on TM3, TM8 and TM10. In the R309 form, a putative hydrogen bond is predicted between R309 and P70/T71 (dashed line); this interaction is absent in the G309 form. Structural alignment, hydrogen-bond prediction and visualization were performed using ChimeraX [59].

Since the predicted protein structure of the transmembrane portion of BicA2 shows a high degree of similarity to the transmembrane portion of the known Na^+^-dependent HCO_3_^−^ transporter BicA1 from *Synechocystis* PCC6803 (BicA1–6803™ [42]; Fig. 3A,B), the HCO_3_^−^ uptake of *E. coli* DH5α expressing BicA2-R309G-1986 was assessed for Na^+^-dependence. BicA2-R309G-1986 HCO_3_^−^ uptake rates increased in response to elevated NaCl concentrations, up to the maximum concentration possible (200 mM NaCl) in the silicone-oil assay (Fig. 2C). In the presence of alternative cations, K^+^ and Li^+^, BicA2-R309G-1986 demonstrated similar HCO_3_^−^ uptake activity as the empty vector control (Fig. 2D), suggesting that Na^+^ is required for HCO_3_^−^ transport. However, when assessed in the presence of various Na^+^ salts where the Na^+^ concentration remained constant, the HCO_3_^−^ uptake activity of BicA2-R309G-1986 was variable (Fig. 2E), suggesting an alternative control mechanism to Na^+^ alone.

The HCO_3_^−^ uptake of *E. coli* DH5α expressing BicA2-R309G-1986 was then assessed in response to a range of NaH^14^CO_3_ concentrations to estimate its affinity for HCO_3_^−^. The concentration of HCO_3_^−^ providing half maximal uptake rate (K_0.5_) was estimated to be 470 ± 42 μM, and a maximum uptake rate (V_max_) estimated on a culture density basis was 812 ± 89 nmol Ci^.^OD_600_^–1.^h^−1^ (Fig. 2F).

Alignments of BicA2 with BicA1 protein sequences that have previously shown to transport HCO_3_^−^ [17, 18, 42] identified a highly conserved GAGATM[G/R]TV motif in transmembrane (TM) helix 10 (Supplementary Figs. S1 and S2). The observed R309G mutation in BicA2–1986 occurs within this motif (Fig. 3C). Both BicA2-R309G-1986 and BicA1 proteins that have demonstrated HCO_3_^−^ transport function to date, have a glycine in this corresponding position (Fig. 3C; Supplementary Fig. S2). We therefore reasoned that the G309 is important for HCO_3_^−^ transport function, and we hypothesized that naturally active forms of BicA2 would carry a glycine residue at this corresponding position. Therefore, in order to identify potentially active native forms of BicA2 in *Prochlorococcus* spp., BLAST searches were performed to identify sequences with high homology to BicA2–1986 (>80% identity) that contained a glycine residue in the conserved TM10 GAGATM**G**TV motif. A single candidate protein (accession #WP_011124397.1) was identified from *P. marinus* CCMP1375 (BicA2–1375) that contained GAGTM**G**TV in TM10 and had high sequence identity (86.8%) to BicA2–1986 (Supplementary Fig. S1; Supplementary Fig. S2).

**Figure 4:**
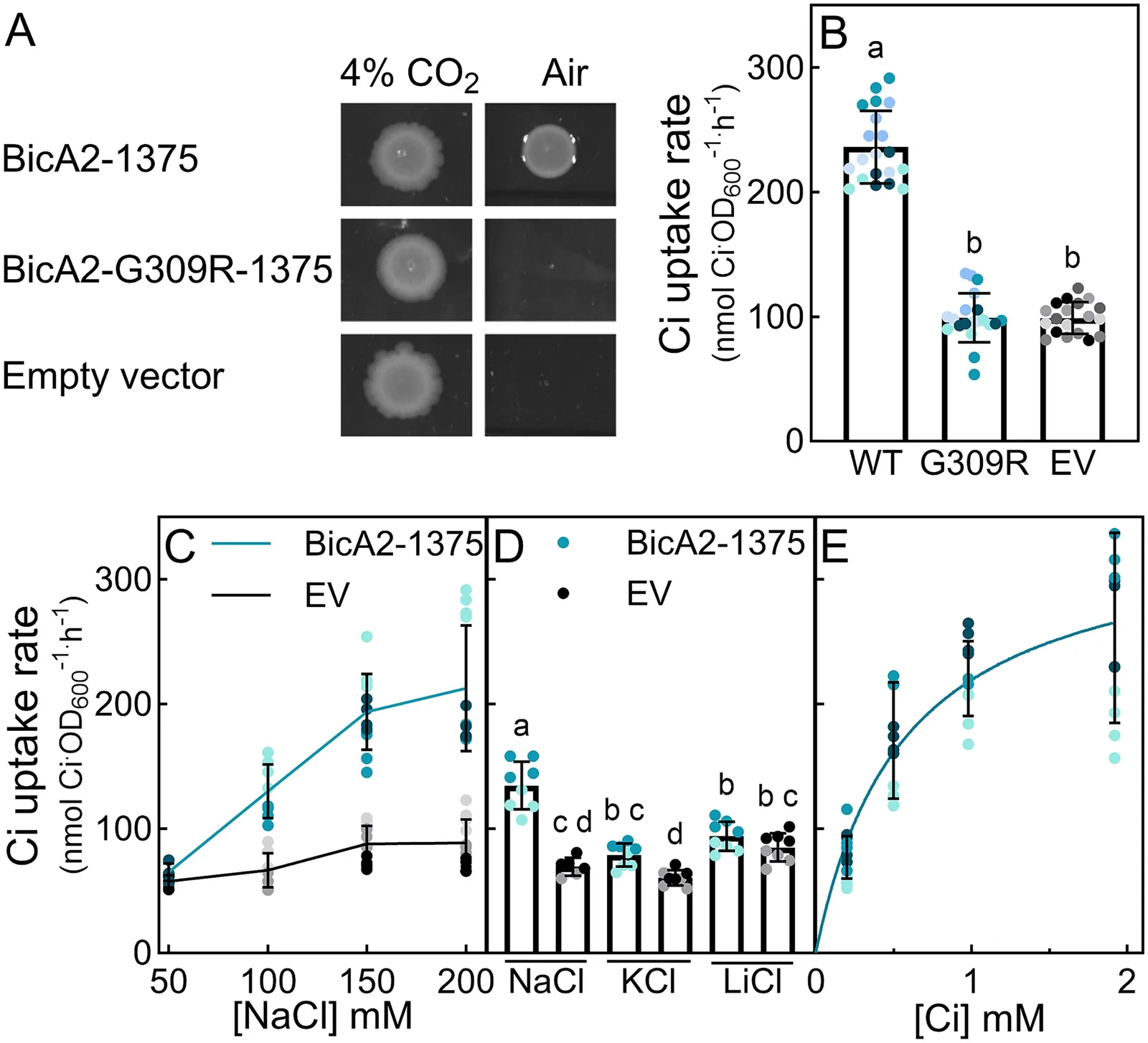
Characterization of BicA2 forms from *Prochlorococcus* CCMP1375 via CAfree *E. coli* complementation and HCO_3_^−^ uptake assays. (A) Cultures of CAfree *E. coli* expressing BicA2–1375, BicA2-G309R-1375 and a negative control (empty vector) were spotted onto selective medium and incubated at air or 4% CO_2_. (B) The HCO_3_^−^ uptake activity of *E. coli* DH5α expressing BicA2–1375 (WT), BicA2-G309R-1375 (G309R) and a negative control (empty vector; EV) was assessed. Assay buffer composition was 20 mM BTP H_2_SO_4_ pH 7.5, 10 mM K_2_HPO_4_, and 200 mM NaCl. Data are presented as means ± SD. n = 20 samples from five biological replicates. (C) HCO_3_^−^ uptake was assessed across a range of NaCl concentrations (50–200 mM). Data are presented as mean ± SD. n = 8–12 from two to three biological replicates. (D) HCO_3_^−^ uptake was assessed in the presence of NaCl, LiCl or KCl. Assay buffer composition was 20 mM BTP H_2_SO_4_ pH 7.5, 10 mM K_2_HPO_4_, 10 mM Na_2_HPO_4_, with relevant salts (150 mM NaCl or 150 mM KCl or 150 mM LiCl). Data are presented as means ± SD, n = 8 from two biological replicates. Different letters indicate significantly different values when assessed using one-way ANOVA (*P* < 0.05). (E) HCO_3_^−^ uptake was assessed over a range of NaHCO_3_ concentrations (0.25–2 mM) to determine the affinity for HCO_3_^−^. For kinetic parameter estimates, the empty vector rates were subtracted prior to non-linear-regression to estimate the Michaelis–Menten constants (Table 1). Data presented as mean ± SD with empty vector rates specific for each condition subtracted. n = 12 from three biological replicates.

CAfree *E. coli* cells expressing BicA2–1375 grew at air levels of CO_2_ (Fig. 4A). However, when compared to BicA2-R309G-1986 (which complemented CAfree in air within 24 h; Fig. 2A), CAfree expressing BicA2–1375 grew slower in air with growth only evident after 48 h (Fig. 4A). HCO_3_^−^ transport by BicA2–1375 was confirmed by HCO_3_^−^ uptake assays (Fig. 4B). Like BicA2-R309G-1986, HCO_3_^−^ uptake by BicA2–1375 was also found to be Na^+^-dependent (Fig. 4C, D) and showed little response to alternative cations (K^+^, Li^+^; Fig. 4D).

Silicone oil centrifugation-filtration assays were used to compare BicA2–1375 and BicA2-R309G-1986 HCO_3_^−^ uptake kinetics. BicA2–1375 showed similar affinity for HCO_3_^−^ to BicA2-R309G-1986, but lower HCO_3_^−^ transport flux (Fig. 4B, E; Table 1). Using estimated *E. coli* cell volumes from a previous study by our group [16], and measured HCO_3_^−^ fluxes (in the presence of 200 mM NaCl and 2 mM Ci), we estimated cellular Ci pool sizes achieved after 30 s uptake assays were between 4.8 and 7.3 mM, and between 3.6 and 6.2 mM for BicA2-R309G-1986 and BicA2–1375, respectively (Table 2).

**Table 1:** Kinetic parameters of BicA2 proteins heterologously expressed in *E. coli* DH5α.

Transporter	K_0.5_ (μM)	V_max_ (nmol Ci OD_600_ ^−1^ h^−1^)
BicA2-R309G-1986	470 ± 42	812 ± 89
BicA2-R309G-mycH6–1986	450	1690
BicA2–1375	670 ± 180	320 ± 94
BicA2-mycH6–1375	790	370

Data for untagged BicA2 forms is presented as mean ± SD of K_0.5_ and V_max_ calculated from individual experiments where Michaelis–Menten non-linear fit had *R^2^* > 0.8. Data for mycH6 tagged BicA2 are from single biological experiments.

**Table 2:** Estimated bicarbonate pool sizes in *E. coli* DH5α expressing BicA2 forms from *Prochlorococcus* spp.

Transporter	Ci pool size (mM)
BicA2-R309G-1986	4.8–7.3
BicA2–1375	3.6–6.2
empty vector	0.5–1.3

Estimated Ci pool sizes achieved in *E. coli* DH5a cells expressing each form of BicA2 transporter in the presence of 200 mM NaCl and 2 mM NaH^14^CO_3._ Cell volumes to enable pool size estimation are based on *E. coli* cell volume estimates of: EV 2–2.5 μl and expressing transporters 1–1.5 μl [16].

We then tested the hypothesis that G309 (GAGATM**G**TV) was important to the activity of BicA2–1375 by generating a mutant form with arginine in this position (hereafter BicA2-G309R-1375), generating the same TM10 motif sequence as the non-functional WT form of BicA2–1986. This mutation abolished growth of CAfree *E. coli* at air levels of CO_2_ (Fig. 4A), and BicA2-G309R-1375 showed no significant HCO_3_^−^ uptake above the empty vector control in *E. coli* DH5α (Fig. 4B).

This outcome led us to explore BicA1 and BicA2 TM10 motif sequence conservation in greater detail through multiple sequence alignment (MSA). Using GAGATMGTV and GAGATMRTV as BLAST queries, we recovered a total of 233 BicA protein sequences with either of these motifs conserved. MSA and phylogenetic tree construction allowed us to identify that the GAGATM**G**TV motif is almost exclusively found in the BicA1 clade, whereas GAGATM**R**TV is almost exclusively found in the BicA2 clade (Supplementary Fig. S1). This analysis also highlighted that, although BicA2–1986 and BicA2–1375 are closely related, BicA2–1375 was the only identified BicA2 protein sequence in our analysis that contained a BicA1-like GAGATM**G**TV motif (Supplementary Fig. S1).

**Figure 5:**
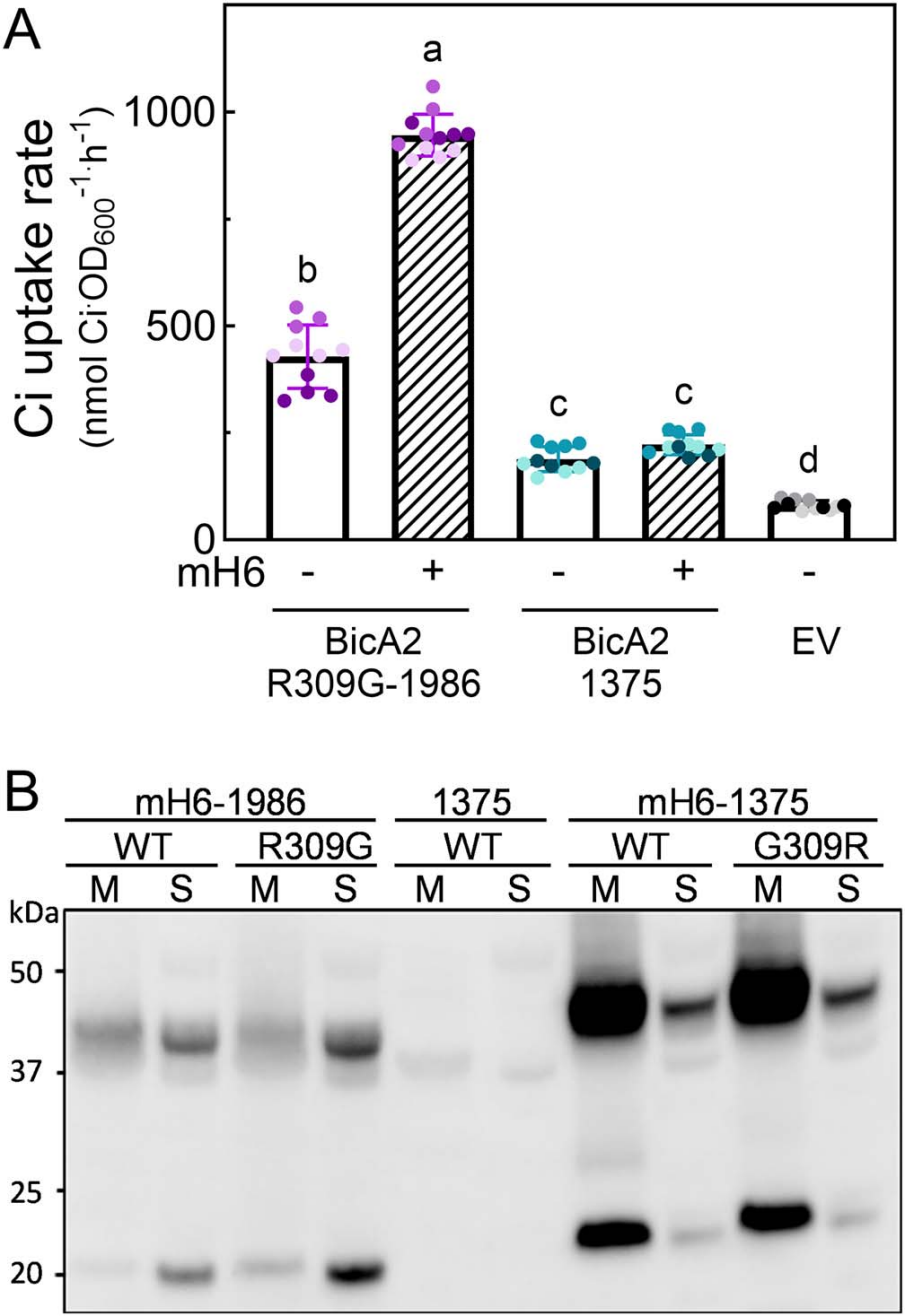
Comparison of protein abundance and HCO_3_^−^ uptake activity of epitope (mycH6)-tagged BicA2 forms. (A) Comparison of HCO_3_^−^ uptake activity of tagged (mH6) and untagged BicA2-R309G-1986 and BicA2–1375, and empty vector (EV) in *E. coli* DH5α. Data are presented as means ± SD, n = 12. Different letters indicate significantly different values when assessed using one-way ANOVA (*P* < 0.05). (B) Western blot analysis of crude soluble (S) and membrane-enriched (M) fractions of *E. coli* DH5α expressing BicA2-mycH6–1986 (WT-mH6–1986), BicA2-R309G-mycH6–1986 (R309G-mH6–1986), BicA2-mycH6–1375 (WT-mH6–1375), BicA2-G309R-mycH6–1375 (G309R-mH6–1375) and BicA2–1375 (WT-1375) to compare the abundance of mycH6-tagged proteins. Samples were loaded on an equivalent culture OD_600_ basis and immunoblots were probed with anti-myc antibody. BicA2–1375 lacking a mycH6 tag is included as a negative control. BicA2-mycH6–1986 bands are observed at ~20 kDa and ~40 kDa, and BicA2-mycH6–1375 bands are observed at ~23 kDa and ~45 kDa.

To identify the sub-cellular location of the BicA2 proteins when expressed in *E. coli* cells, and to compare the relative expression of mutant and WT BicA2 forms from *Prochlorococcus* CCMP1986 and CCMP1375, each protein was C-terminally tagged with a mycH6 epitope using a glycine and serine linker (GS-EQKLISEEDL-HHHHHH). The HCO_3_^−^ uptake activity of tagged forms of BicA2 was not impeded (Fig. 5). Indeed, the tagged form of BicA2-R309G-1986 had approximately twice the HCO_3_^−^ uptake rate as its untagged counterpart when measured based on cell density (Fig. 5A). This could be due to improved stability of BicA2-R309G-mycH6–1986 rather than a modulation of protein function.

Using this epitope tag as a semi-quantitative tool, we assessed relative protein abundance by immunoblotting on soluble (cytoplasmic) and membrane-enriched *E. coli* DH5α cell fractions. We found that the majority of BicA2-R309G-mycH6–1986 was present in the soluble fraction (Fig. 5B), suggesting minimal insertion and stability of this protein into the cell membrane. Furthermore, there was a large disparity between the relative quantities of BicA2-R309G-mycH6–1986 and BicA2-mycH6–1375 expressed in *E. coli* DH5α, indicating that, on a per-protein basis, BicA2-R309G-1986 is the more active form of the pair, or possibly less prone to protease breakdown at the C-terminus, as suggested by the lower molecular weight immunoreactive bands (Fig. 5B). In addition, despite the relatively low Ci uptake activity of BicA2-mycH6–1375, there appeared to be significantly greater quantities of this protein in the membrane fraction of *E. coli* cells (Fig. 5B).

## Discussion

The dominant marine cyanobacterial genus *Prochlorococcus* [4] plays a critical role in global carbon fixation [2, 45], yet its mechanism to take up Ci from the aquatic environment has, to date, been speculative. *Prochlorococcus* spp. carry genes for distant relatives of the previously characterized HCO_3_^−^ transporters, SbtA1 and BicA1, namely SbtA2 and BicA2 [19, 26, 27]. However, the *Prochlorococcus* homologues represent distinct sub-families (Fig. 1) [19, 26, 27] with potentially different modes of operation. Whereas SbtA2 from *Synechococcus* spp. have shown HCO_3_^−^ transport activity [19], no verified bicarbonate uptake function has been reported for either SbtA2 or BicA2 proteins from *Prochlorococcus* spp.

Here we demonstrate HCO_3_^−^ transport by BicA2 homologues from *Prochlorococcus*, and identification of a conserved sequence motif in TM10 across BicA2 homologues. The active BicA2 proteins display low affinity, Na^+^-dependent HCO_3_^−^ transport (Fig. 2; Fig. 4; Table 1), providing evidence for HCO_3_^−^ transport in the ecologically important *Prochlorococcus* genus.

### HCO_3_^−^ transporters and their heterologous expression

The BicA class of transporters are part of the broader SLC26 sulfate transporter superfamily [17]. Proteins in this family typically consists of two domains, the N-terminal membrane transport domain and a regulatory sulfate transporter anti-sigma antagonist (STAS) domain constituting the C-terminal portion of the protein [42, 46]. Evidence from eukaryotic SLC26 transporters indicates that the STAS domain functions as a conserved cytoplasmic regulatory domain that links transporter assembly and stability with regulatory protein interactions and anion transport activity [47]. Like SbtA1 and SbtA2 [19], BicA1 and BicA2 appear to belong to distinct subfamilies (Fig. 1) [26]. BicA1 has been characterized to be a Na^+^-dependent HCO_3_^−^ transporter with low substrate affinity, and a relatively high flux compared to SbtA1 and BCT1 [17, 48]. Function of BicA1 has been described in its native host, *Picosynechococcus* PCC7002 [17], as well as when heterologously expressed in cyanobacteria (*Synechococcus* PCC7942) [17] (*Synechocystis* PCC6803) [18], and the chemoautotrophic bacterium *Cupriavidus necator* H16 [49]. However, its functional characteristics have been elusive in *E. coli*, where function appears to be locked in an inactive state [16].

There has been no specifically observed function of any BicA2 forms to date, except for the physiological analysis of *Prochlorococcus* CCMP1986 (also known as *Prochlorococcus* MED4) [29] which has two proteins related to BicA1, including one closely related BicA2 form and a more distant relative (Fig. 1) [27, 29,], and an SbtA2 [19, 26]. After initial screening in *E. coli* CAfree showed no function, an evolved form of BicA2–1986 with a mutation in a conserved TM10 sequence motif, GAGATM**G**TV, allowed us to identify a sequence relative (BicA2–1375) that was functional in CAfree in its native form. This finding enabled identification of a conserved motif that plays a role in BicA2 function.

Functional characterization of the two active BicA2 forms (BicA2–1375 and BicA2-R309G-1986) showed that they both had K_0.5_ for HCO_3_^−^ of ~470–670 μM (Fig. 2F; Table 1). This is low affinity compared to estimates of K_0.5_ for HCO_3_^−^ for SbtA1 and SbtA2 of <100–190 μM, and ~ 150 μM respectively when measured in *E. coli* [16, 19]. Even though the affinity for HCO_3_^−^ of these BicA2 proteins is relatively low, these values still lie below the HCO_3_^−^ concentrations available in marine environments (~2 mM HCO_3_^−^) [50]. This highlights the potential of BicA2 to function effectively at physiologically relevant conditions. In addition, estimated cellular Ci pool sizes of up to 6.2 and 7.3 mM (BicA2–1375 and BicA2-R309G-1986 respectively; Table 2) were achieved in *E. coli* when 2 mM Ci was supplied in uptake assays. Noting this is an artificial system, these pool sizes approach the level required to achieve saturation of CO_2_ fixation (>200 μM CO_2_) of the Form-IA Rubisco found in these organisms within their α-carboxysomes [51, 52] through equilibration of the cellular HCO_3_^−^ pool with CO_2_ inside the carboxysome [7]. A previous study [30] suggested *P. marinus* MIT9313 likely has at least a high affinity HCO_3_^−^ transporter (possibly SbtA2). Other work [29] provided evidence that the relatively simple CCM of *Prochlorococcus* CCMP1986 (consisting of at least one BicA2 protein and an SbtA2 as the only Ci uptake systems; Fig. 1) operates efficiently. Internal Ci pools sizes in *Prochlorococcus* CCMP1986 were measured to be ~15 mM in the presence of 2 mM external Ci [29]. Based on the observed HCO_3_^−^ uptake kinetics, this study suggested that a BicA-like transporter was the dominant contributor to bulk HCO_3_^−^ uptake under the conditions examined, although they suggested that SbtA2 may also be active [29]. Neither *bicA2* nor *sbtA2* transcript abundance changed significantly in response to CO_2_ availability, suggesting both transporters may be constitutively expressed under the conditions examined [29]. The data presented here demonstrate that BicA2 proteins are capable of mediating Ci uptake in *E. coli* and support the hypothesis that BicA2 contributes to Ci acquisition in *Prochlorococcus*, providing a pathway for carbon influx to support carbon fixation in this globally significant phototroph. However, the quantitative contribution of BicA2 to cellular bicarbonate uptake in native *Prochlorococcus* cells remains to be determined.

When the Na^+^-dependence of BicA2-R309G-1986 was assessed in *E. coli*, the highest HCO_3_^−^ uptake rates were evident in the presence of NaCl, but not when equimolar quantities of Na^+^ were supplied as Na_2_SO_4_ or NaNO_3_ (Fig. 2E). This suggests a potentially undescribed mechanism of action, and may indicate a parallel Na^+^-driven Cl^−^/HCO_3_^−^ exchange mechanism, as seen in some HCO_3_^−^ transporters of the solute carrier 4 (SLC4) family [53]. From the experimental analysis here, we cannot confirm a dependence on Cl^−^. Alternatively, reduced uptake in the presence of nitrate or sulfate may reflect competition by these oxyanions for interaction with the transporter. Further assessment of this observation is required to confirm the mode of action of BicA2 members, which could potentially be evaluated in *Xenopus* oocytes [54, 55]. Whether this apparent preference for NaCl is a general feature of BicA2 proteins or specific to BicA2-R309G-1986 remains unclear; comparable experiments were not performed for BicA2–1375 because the focus here was on the mechanistic properties of BicA2-R309G-1986.

### Key amino acid sequences for BicA2 activity

Structural studies have [42] identified amino acid residues involved in putative HCO_3_^−^ binding sites in BicA1 from *Synechocystis* PCC6803. Alphafold3-generated structural alignments [56] reveal a high degree of shared tertiary elements between BicA1 and BicA2 (Fig. 3A). Additionally, BicA1 and BicA2 proteins that have shown HCO_3_^−^ transport function [this study; 17, 18, 42] demonstrate conservation of the proposed HCO_3_^−^ binding site amino acids and the GAGATM**G**TV motif in TM10 (Supplementary Fig. S2). These identify potentially critical sequence elements across all BicA proteins that are essential for function. The sequential A and T residues which we find in the GAG**AT**MGTV motif are indicated to be important in HCO_3_^−^ and metal binding [42]. The BicA1–6803 G_304_ residue (GAGATM**G**TV) is also likely involved in coordinating Na^+^ [42]. Additionally, the same study identified that some Cl^—^coupled HCO_3_^−^ transporters (e.g. some SLC4 and SL26 family Cl^−^/HCO_3_^−^ exchangers) contain an arginine at the amino acid position corresponding to position 304 of BicA1–6803 (i.e. GAGATM**R**TV), whereas some Na^+^-coupled HCO_3_^−^ transporters (e.g. SLC4 family Na^+^/HCO_3_^−^ cotransporters and BicA1) contained a glycine at this position (i.e. GAGATM**G**TV), providing further evidence for different modes of action between BicA1 and BicA2.

The role of R_309_ in the GAGATM**R**TV motif of native BicA2–1986 remains unclear, despite this sequence being conserved across BicA2 proteins (Supplementary Fig. S1). One possibility is that transporters with this motif require activation and are normally inactive. In contrast, the R309G mutation may produce an “always-active” form, bypassing such regulation. Structural predictions suggest that R_309_ forms H-bonds with P_70_/T_71_ in the WT protein, interactions absent from BicA2-R309G-1986 (Fig. 3C), potentially increasing conformational flexibility and enabling activity without activation partners.

We propose that canonical BicA2 proteins bearing the GAGATM**R**TV motif are intrinsically capable of HCO_3_^−^ transport but are normally constrained by regulatory or activation requirements that are not met in *E. coli*. Under this model, adaptive laboratory evolution or rare natural substitutions that replace the TM10 arginine with glycine may generate an “always-active” transporter, thereby bypassing these constraints. BicA2–1375 appears to be an outlier in the BicA2 clade, having a GAGATM**G**TV motif in TM10 when all its close relatives have a GAGATM**R**TV motif in this region (Supplementary Fig. S1), yet it is functional whereas BicA2–1986 (having the canonical motif sequence for this clade) is not (Fig. 2). It is possible that BicA2–1375 has evolved toward a canonical BicA1-type protein. Whereas BicA2–1375 has lower substrate affinity than functional BicA2–1986 forms (Table 1), BicA2–1375 appears to be capable of achieving intercellular Ci pool sizes (albeit in *E. coli*) capable of supporting CCM function (Table 2; [51]). It is conceivable that stable growth conditions for *Prochlorococcus* CCMP1375 in lab culture prior to its genomic sequencing [5], could lead to stable populations with this genotype.

## Conclusion

*Prochlorococcus* spp. are major contributors to marine primary productivity [2], yet direct evidence for the HCO_3_^−^ transport pathways underpinning their photosynthesis has been lacking. Here, we demonstrate that BicA2 proteins from *Prochlorococcus* function as a Na^+^-dependent HCO_3_^−^ transporters when expressed in *E. coli*. Together with the absence of alternative transport systems and its similarity to BicA1, our results support the hypothesis that BicA2 functions as a HCO_3_^−^ transporter in *Prochlorococcus* and may contribute to the Ci uptake required to sustain carboxysomal CO_2_ supply [29], although confirmation of its physiological role in native cells will require genetic or physiological analyses in *Prochlorococcus*. Our findings further suggest that BicA2 proteins are intrinsically capable of transport, with the conserved arginine in the TM10 GAGATMRTV motif imposing an inactive or regulated state that can be relieved by substitution to glycine. We propose that BicA2 operates alongside the higher-affinity SbtA2 [19] to enable efficient CCM function in this important contributor to global primary productivity.

## Supplementary Material

Web_Material_wrag222

## Data Availability

The primary data supporting this study are publicly available at https://data.mendeley.com/datasets/g2f8ghkrnj/1 and are available from the corresponding author upon request. Additional data reported in this paper are presented in the Supplementary Data.

## Abbreviations

aTc, anhydrotetracycline hydrochloride; BicA, bicarbonate transporter A; CA, carbonic anhydrase; CA free, specialized *E. coli* strain that lacks CAs; CCM, CO_2_-concentrating mechanism; CO_2_, carbon dioxide; Ci, inorganic carbon (CO_2_ and HCO_3_^−^); HCO_3_^−^, bicarbonate ion; IPTG, isopropyl β-D-1-thiogalactopyranoside; LB, Luria-Bertani; Na^+^, sodium ion; WT, wild-type.
