# Supplementary material for: Heterologous expression in E. coli reveals that bicarbonate transporter BicA2 mediates carbon uptake in marine Prochlorococcus spp
Source: ISME J. 2026 Aug 27;20(1):wrag222. doi: 10.1093/ismejo/wrag222 (PMC13588710; doi:10.1093/ismejo/wrag222)
Supplement: Supplementary file 1 [file web_material_wrag222.zip › Supplementary figures.pdf]

Supplementary Information for

Heterologous expression in *E. coli* reveals that bicarbonate transporter BicA2 mediates carbon uptake in marine *Prochlorococcus* spp.

**Authors**:	**ORCID**	**ResearcherID**
Loraine M. Rourke [1,2]	0000-0002-2600-8073	OGM-9247-2025
Caitlin S. Byrt [1]	0000-0001-8549-2873	I-9785-2018
G. Dean Price [1]	0000-0001-5906-4912	C-9505-2009
Benedict M. Long [1,3,4*]	0000-0002-4616-2967	B-3799-2008

Addresses:

[1]Plant Science Division, Research School of Biology, 134 Linnaeus Way, Australian National University, Acton, ACT, Australia, 2601 Current address: [2]Plant SynBio Australia, Plant Science Division, Research School of Biology, 134 Linnaeus Way, Australian National University, Acton, ACT, Australia, 2601,[3]Discipline of Biological Sciences, and [4]ARC Centre of Excellence in Synthetic Biology, School of Science, University Drive, The University of Newcastle, Callaghan, NSW, Australia, 2308

*To whom correspondence should be addressed: ben.long@newcastle.edu.au

This supplementary information includes:

7 pages

1 table

3 figures

Supplementary Table:

**Table S1 Primers used for amplification of *bicA2-1986***	
1986_BicA2R_pt1	AAGAAGACCGGACACCACAGAATATGGAACAAGCGTT
1986_BicA2F_pt1	AAGAAGACAACTCAAGGTTTGGAAATAATTAATGGATTTAATC
1986_BicA2R_pt2	AAGAAGACAACTCGCGAACCGGCTAATTCATTTAAAGCAGCTTC
1986_BicA2F_pt2	AAGAAGACGGTGTCCGGATTTATGTCTGGCATAGGAG

Each primer pair has been designed to enable domestication of the *Prochlorococcus marinus* CCMP1986 BicA2 sequence for Loop assembly [1], a silent change (GTC to GTG) removing a BsaI site within the coding sequence. Primers are designed 5’ to 3’.

Supplementary Figures

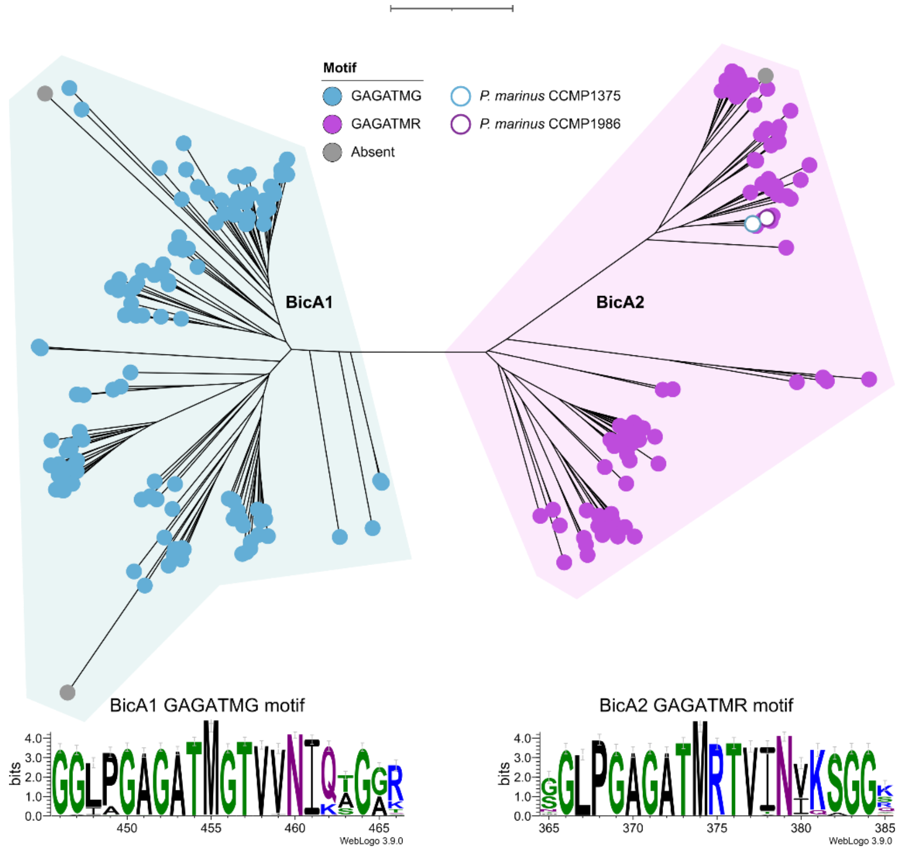

Supplementary Figure S1: GAGATM[G/R]TV motif conservation in transmembrane domain 10 (TM10) of BicA1 and BicA2 clades.
Identification of conserved GAGATMGTV or GAGATMRTV motifs across BicA forms highlights that the GAGTMGTV motif is almost exclusively found in the BicA1 clade, while GAGTMRTV is almost exclusively found in the BicA2 clade. The non-functional *Prochlorococcus marinus* CCMP1986 BicA2 form (BicA2-1986) has a GAGATMRTV motif in TM10 and is among relatives which also contain the GAGATMRTV motif. It's mutation to a BicA1-like GAGATMGTV sequence (BicA2-R309G-1986; Figure 2) enabled function. The natively functional BicA2 from *P. marinus* CCMP1375 (BicA2-1375) contains a GAGATMGTV motif in TM10 and its mutation to a BicA2-like GAGATMRTV motif (BicA2-G309-R-1375; Figure 4) leads to loss of function. Sequence alignment performed using MAFFT 7.526 [5] from a curated BicA dataset containing 223 protein sequences (Supplementary files) with default parameters and 1000 bootstrap replicates. Tree graphics were generated using iTol [6] identifying all sequences with a GAGATMGTV motif in blue, all GAGATMRTV motifs in purple and sequences with no identified GAGAMT[G/R]TV motif in grey. Tree scale is 0.1 substitutions per amino acid. Sequence logos are based on separate alignments of the BicA1 and BicA2 sequences in the curated sequence dataset. Residues coloured by chemistry, and logos were generated using WebLogo3.

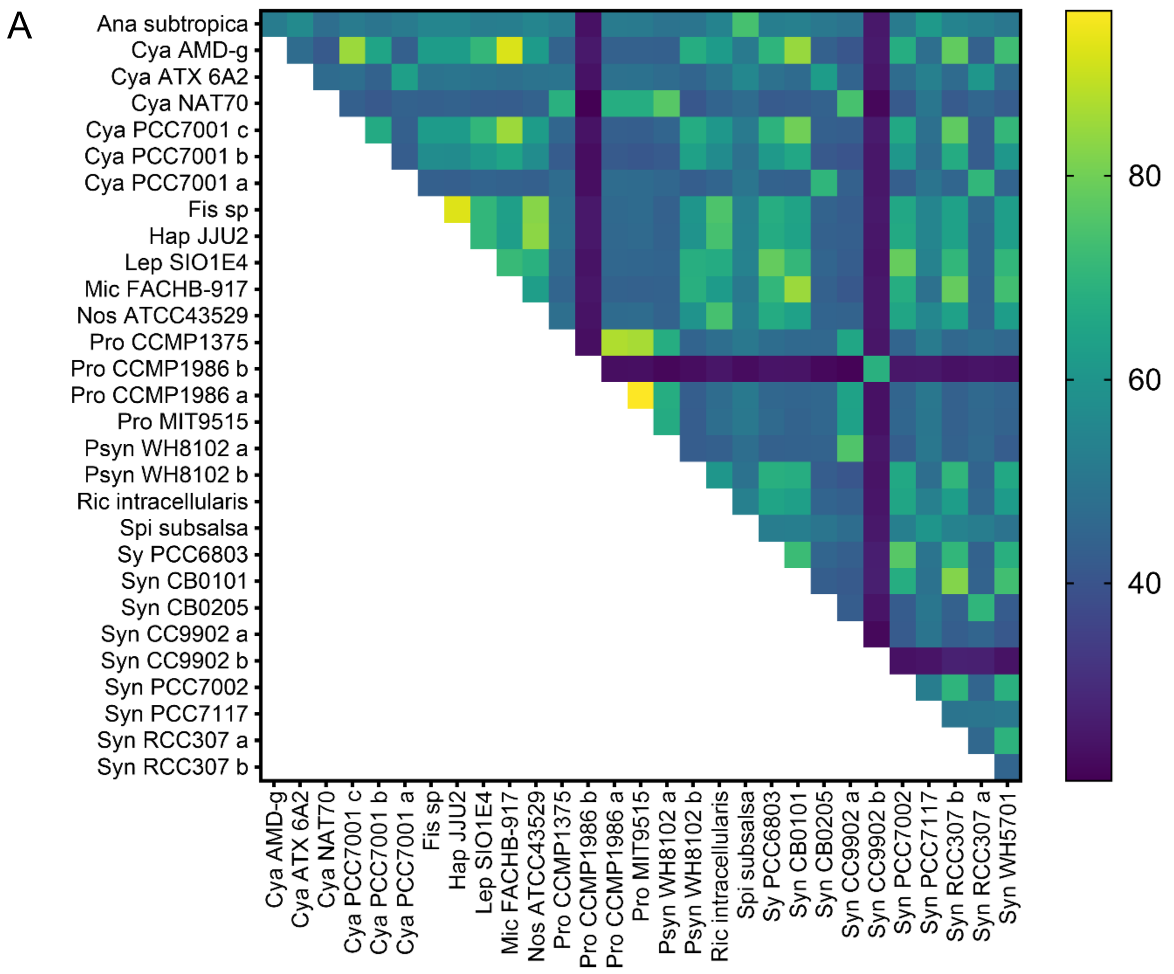
A
Ana subtropica
Cya AMD-g
Cya ATX 6A2
Cya NAT70
Cya PCC7001 c
Cya PCC7001 b
Cya PCC7001 a
Fis sp
Hap JJU2
Lep SIO1E4
Mic FACHB-917
Nos ATCC43529
Pro CCMP1375
Pro CCMP1986 b
Pro CCMP1986 a
Pro MIT9515
Psyn WH8102 a
Psyn WH8102 b
Ric intracellularis
Spi subsalsa
Sy PCC6803
Syn CB0101
Syn CB0205
Syn CC9902 a
Syn CC9902 b
Syn PCC7002
Syn PCC7117
Syn RCC307 a
Syn RCC307 b
Syn WH5701
80
60
40

B

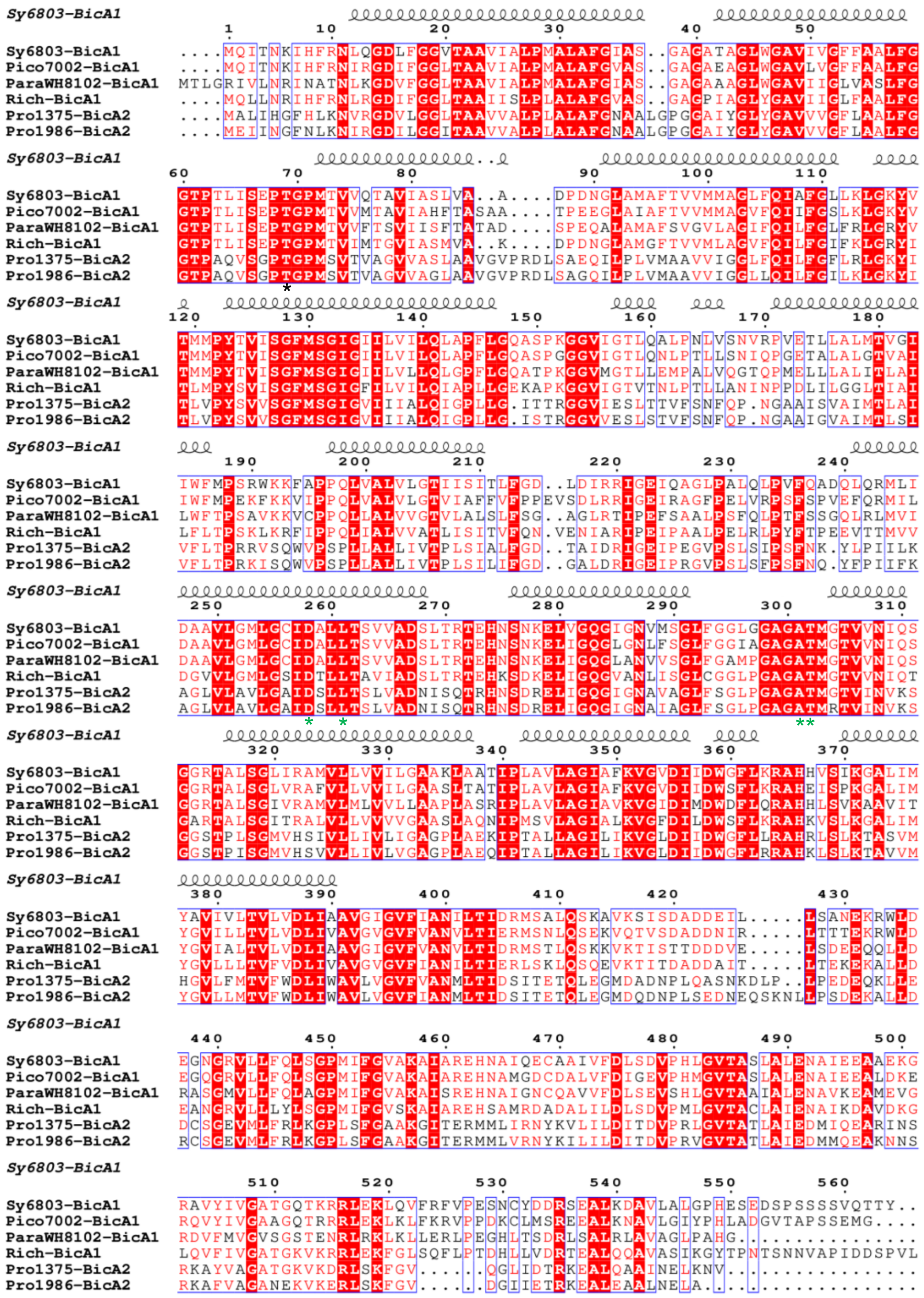

Supplementary Figure S2: Comparison of select BicA1 and BicA2 protein sequences demonstrate key sequence differences.

A) Heat map showing the % similarity of select members of the BicA family in Figure 1. The higher the number the more similar the sequence. Psyn: *Parasynechococcus*, Syn: *Synechococcus*, Pro: *Prochlorococcus*, Cya: *Cyanobium*, Mic: *Microcystis*, Lep:

Leptolyngbya, Fis: *Fischerella*, Hap: *Hapalosiphonaceae*, Nos: *Nostoc*, Sy: *Synechocystis*, Ana: *Anabaena*, Spi: *Spirulina*, Ric: *Richelia*, Pico: *Picosynechococcus.* Alignment performed by Geneious Prime and graphed using GraphPad. **B)** Alignment of the amino acid sequences of select members of the BicA family. See Figure 1 for the phylogeny tree. The structure for the transmembrane portion of *Synechocystis* PCC6803 BicA1 [2], was used for the structural label, alignment performed by MultAlin [3] and graphics by ESPript [4]. Asterisks (*) indicate the putative HCO_3^- binding residues. The GAGATM[G/R]TV motif of transmembrane domain 10 (TM10) identified in this study begins at position 298 relative to the *Synechocystis* PCC6803 BicA1 sequence.

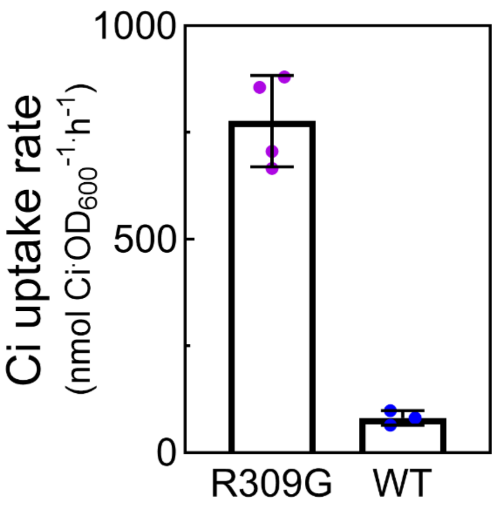

Supplementary Figure S3: **Bicarbonate uptake by mutant BicA2-R309G-1986 after re-cloning of the evolved gene**. After recovery of mutant CAfree *E. coli* cells carrying *bicA2-1986* capable of growth at 0.04% CO_2 (v/v) in air, isolated plasmid was used as the template for PCR and the evolved *bicA2* was assembled with pCk1 vector*, lacI^q-trc* promoter and rrnB terminator components to create the expression plasmid (Materials and Methods). This re-cloned gene was introduced into *E. coli* DH5α cells and $NaH^{14}CO_3$ uptake rates were carried out as described in the main text. Compared here are the uptake rates of cells carrying the mutated BicA2-R309G-1986, and its wildtype counterpart (WT; BicA2-1986), both expressed from the original pCk1 vector. All subsequent experiments were carried out using this re-cloned form of *bicA2-R309G-1986*.

References

1. Pollak B, Cerda A, Delmans M, et al. Loop assembly: a simple and open system for recursive fabrication of DNA circuits. *New Phytologist*. Apr 2019;222(1):628–640. doi:10.1111/nph.15625
2. Wang C, Sun B, Zhang X, et al. Structural mechanism of the active bicarbonate transporter from cyanobacteria. Article. *Nature Plants*. Nov 2019;5(11):1184–1193. doi:10.1038/s41477-019-0538-1
3. Corpet F. Multiple sequence alignment with hierarchical clustering. *Nucleic Acids Res*. Nov 25 1988;16(22):10881–90. doi:10.1093/nar/16.22.10881
4. Robert X, Gouet P. Deciphering key features in protein structures with the new ENDscript server. *Nucleic Acids Res*. 2014;42(W1):W320–W324. doi:10.1093/nar/gku316
5. Katoh K, Rozewicki J, Yamada KD. MAFFT online service: multiple sequence alignment, interactive sequence choice and visualization. *Brief Bioinform*. 2017;20(4):1160–1166. doi:10.1093/bib/bbx108
6. Letunic I, Bork P. Interactive Tree of Life (iTOL) v6: recent updates to the phylogenetic tree display and annotation tool. *Nucleic Acids Res*. 2024;52(W1):W78–W82. doi:10.1093/nar/gkae268
